# Decreased functional connectivity is associated with increased levels of Cerebral Spinal Fluid soluble-PDGFRβ, a marker of blood brain barrier breakdown, in older adults

**DOI:** 10.21203/rs.3.rs-2644974/v1

**Published:** 2023-03-07

**Authors:** Joey Annette Contreras, Kimiko Fujisaki, Nancy Ortega, Giuseppe Barisano, Abhay Sagare, Ioannis Pappas, Helena Chui, John M Ringman, Elizabeth B Joe, Berislav Zlokovic, Arthur W Toga, Judy Pa

**Affiliations:** University of California; University of Southern California; University of California; University of Southern California; University of Southern California; University of Southern California; University of Southern California; University of Southern California; University of Southern California; University of Southern California; University of Southern California; University of California, San Diego

**Keywords:** resting-state functional magnetic resonance imaging (rsfMRI), default mode network (DMN), BBB breakdown, soluble platelet-derived growth factor receptor-β (sPDGFRβ), cognitive impairment

## Abstract

Resting-state functional connectivity (FC) is suggested to be cross-sectionally associated with both vascular burden and Alzheimer’s disease (AD) pathology. For instance, studies in pre-clinical AD subjects have shown increases of cerebral spinal fluid soluble platelet-derived growth factor receptor-β (CSF sPDGFRβ, a marker of BBB breakdown) but have not demonstrated if this vascular impairment affects neuronal dysfunction. It’s possible that increased levels of sPDGFRβ in the CSF may correlate with impaired FC in metabolically demanding brain regions (i.e. Default Mode Network, DMN). Our study aimed to investigate the relationship between these two markers in older individuals that were cognitively normal and had cognitive impairment. Eighty-nine older adults without dementia from the University of Southern California were selected from a larger cohort. Region of interest (ROI) to ROI analyses were conducted using DMN seed regions. Linear regression models measured significant associations between BOLD FC strength among seed-target regions and sPDGFRβ values, while covarying for age and sex. Comparison of a composite ROI created by averaging FC values between seed and all target regions among cognitively normal and impaired individuals was also examined. Using CSF sPDGFRβ as a biomarker of BBB breakdown, we report that increased breakdown correlated with decreased functional connectivity in DMN areas, specifically the PCC while the hippocampus exhibited an interaction effect using CDR score. We conclude that BBB breakdown as measured by CSF sPDGFRβ affects neural networks resulting in decreased functional connections that leads to cognitive dysfunction.

## Introduction

Although beta-amyloid and phosphorylated tau are the characteristic neuropathological hallmarks of Alzheimer’s disease, cerebrovascular dysfunction and vascular pathology have been reported to serve an important role in the onset and progression of Alzheimer’s disease (AD). This is the premise of the two-hit vascular hypothesis for AD (Zlokovic 2011), such that the first hit proposes an initial insult that causes damage to blood vessels leading to blood brain barrier (BBB) dysfunction. Then, the subsequent hit is the resulting diminished brain perfusion which leads to increased AD pathology in the brain and ultimate neuronal loss. Patients with AD also show disruptions in functional connectivity, especially within default mode network (DMN) regions (Jones et al., 2011). The DMN is a collection of brain regions that exhibit synchronized low temporal frequency blood oxygen level dependent (BOLD) activity measured using resting state functional magnetic resonance imaging (rsfMRI). Although the brain regions are spatially segregated, they are intrinsically coactivated and deactivated across time and considered to be functionally connected under task free conditions. The DMN consists of the bilateral parietal lobes, posterior cingulate cortex, medial prefrontal cortex (MPFC), and hippocampi (Raichle et al., 2001). Decreased functional connectivity among DMN regions has been associated with the neurodegeneration and cognitive decline related to AD (Staffaroni et al., 2018; Hedden et al., 2009; Zhou et al., 2010), possibly for the DMN’s functional role in autobiographic memory, future thinking, and introspection (Buckner and Carroll, 2007; Buckner et al, 2008).

BBB breakdown has been observed in DMN brain regions in individuals with mild cognitive impairment (MCI) (Montagne et al., 2016; Nation et al., 2019; Barisano et al., 2022). For instance, using dynamic contrast-enhanced MRI (DCE-MRI), increased CNS BBB permeability was observed in the hippocampi of individuals with MCI compared to age-matched cognitively unimpaired older adults. This finding correlated with increased levels of soluble platelet-derived growth factor receptor-β (sPDGFRβ) suggesting that cerebrospinal fluid (CSF) sPDGFRβ may be a biomarker of BBB breakdown via pericyte injury. Because regions within the DMN seem to serve as both connectivity “hubs” and the site of pathological insult within AD, and because the link between DMN functional connectivity and BBB breakdown has not been investigated despite the relevance to AD risk and pathogenesis, we investigated the relationship between vascular and functional activity changes. In this study, the association between CSF sPDGFRβ and DMN functional connectivity was evaluated to better characterize the link between two early indicators of AD risk.

## Methods

### Participants

Participants were recruited through the University of Southern California Alzheimer’s Disease Research Center (ADRC) in Los Angeles, CA. The study and procedures were approved by the Institutional Review Board indicating compliance with all ethical regulations, and informed consent was obtained from all participants prior to study enrollment. All participants underwent neurological and neuropsychological evaluations performed using the Uniform Data Set (UDS) and additional neuropsychological tests, as described below. Eighty-nine participants were included based on availability of T1-weighted MPRAGE scan, rsfMRI scan, Clinical Dementia Rating (CDR) score, and CSF sPDGFRβ biomarker data that was collected within 90 days of the MRI scan date. All biomarker assays and quantitative MRI scans were conducted by investigators blinded to the clinical status of the participant.

### Inclusion/Exclusion Criteria

Participants were included if they were at least 45 years of age, were cognitively unimpaired or exhibited early cognitive dysfunction, and had no current or prior history of any neurological or psychiatric conditions that could explain any observed cognitive impairment, including organ failure, brain tumors, epilepsy, hydrocephalus, schizophrenia, major depression, Parkinson’s disease, Lewy body dementia or frontotemporal dementia. Participants could not have current contraindications to MRI and use medications that might explain any observed cognitive impairment.

### Clinical Dementia Rating (CDR)

Clinical Dementia Rating (CDR) assessments followed the standardized process for CDR interviews. Participants underwent clinical interview, including health history, and a physical exam. Knowledgeable informants were also interviewed. Participants who had a CDR score of 0 were categorized as cognitively unimpaired and those with a score of greater than 0 were categorized as cognitively impaired for the purposes of this study.

### Lumbar Puncture and Molecular Biomarkers in the Cerebrospinal Fluid (CSF) Assays

Participants underwent lumbar puncture in the morning after an overnight fast. CSF was collected in polypropylene tubes, processed (centrifuged at 2000; relative centrifugal force (RCF) for 10 minutes at 4°C), aliquoted into polypropylene tubes and stored at − 80°C until the time of assay.

### APOE Genotyping

APOE genotyping was performed as described in Nation et al. (2019). Participants with at least one copy of the E4 allele were considered APOE4 carriers. There were no E4 homozygous carriers.

### Quantitative Western Blotting of sPDGFRβ

Quantitative Western blot analysis was used to determine CSF levels of sPDGFRβ in human CSF (ng/mL). Standard curves were generated using recombinant human PDGFRβ (Cat. No. 385-PR-100/CF, R&D Systems, Minneapolis, MN) (as described in Nation et al., 2019).

### MRI Data Acquisition, Preprocessing and Analysis

**Figure 1:**
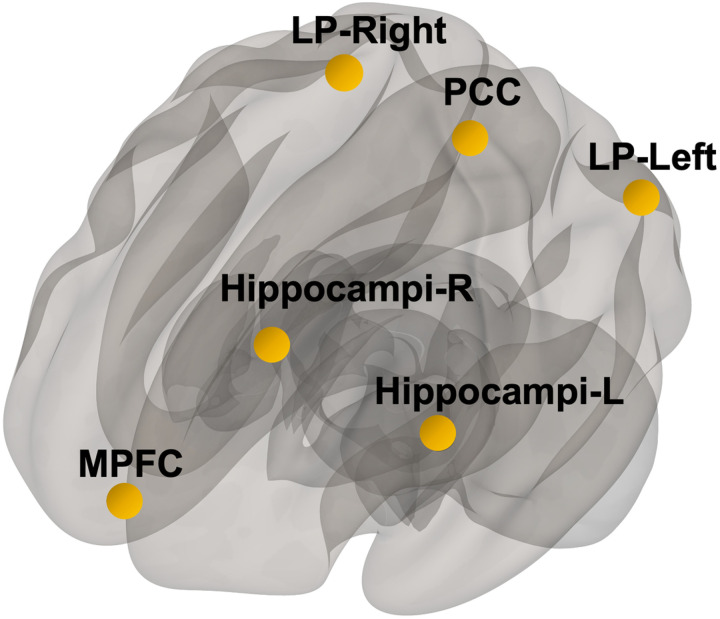
Seed Regions. Default mode network brain regions were selected as seed regions which consisted of medial prefrontal cortex (mPFC), posterior cingulate cortex (PCC), bilateral parietal brain regions (LP-left, LP-right), and bilateral hippocampi regions.

rsfMRI images were preprocessed using the CONN-toolbox v18a (Whitfield-Gabrieli and Nieto-Castanon, 2012) in SPM12 for data analysis. The preprocessing pipeline of the functional images consisted of motion correction, coregistration to structural images, spatial normalization to the Montreal Neurological Institute (MNI) template, smoothing with a 5mm full-width at half-maximum Gaussian kernel, and band-pass filtering of 0.009–0.1 Hz. After preprocessing, the CompCor strategy (Behzadi et al., 2007) was implemented to account for white matter and CSF noise using principal component analysis. The analyses did not include global signal regression to avoid potential false anticorrelations (Murphy et al., 2009). Motion parameters, cerebrospinal fluid, and white matter were included in the model and considered as variables of no interest. The mean BOLD signal time course was then extracted from every ROI predefined by the Harvard-Oxford atlas and resting state networks as defined by Shen and colleagues (Shen et al., 2013), except for cerebellar and primary sensory areas (limiting the number of ROIs to 138). Pearson’s correlation coefficients were calculated for all pairwise comparisons between ROIs making this an ROI to ROI analysis. Linear regression was used to compute the correlation between all seed and target ROIs functional connectivity (FC) strength and sPDGFRβ. DMN regions of bilateral parietal lobes, posterior cingulate cortex, medial prefrontal cortex, and hippocampi were used as seed regions (Fig. 1).

**Figure 2:**
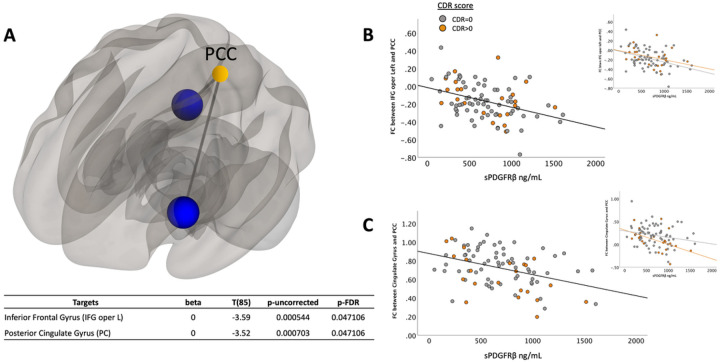
sPDGFRβ Values Negatively Correlated with Resting-State Functional Connectivity using PCC as seed region. Linear regression analysis revealed the functional connectivity between PCC and both the left inferior frontal (R^2^=0.13, *t*(85)=−3.59, p=0.047) and posterior cingulate gyrus (R^2^=0.14, *t*(85)=−3.52, p=0.047) was lower as sPDGFRβ values increased displayed on a glass brain shown in Panel A. Data were plotted to visualize differences between cognitively normal individuals (colored in grey) versus those with mild cognitive impairment (colored in orange). Panel B shows functional connectivity values between PCC and inferior frontal gyrus on the y axis and its relationship to sPDGFRβ values on the x axis. Panel C plots functional connectivity between PCC and the posterior cingulate gyrus. Age and sex were used as covariates.

**Figure 3:**
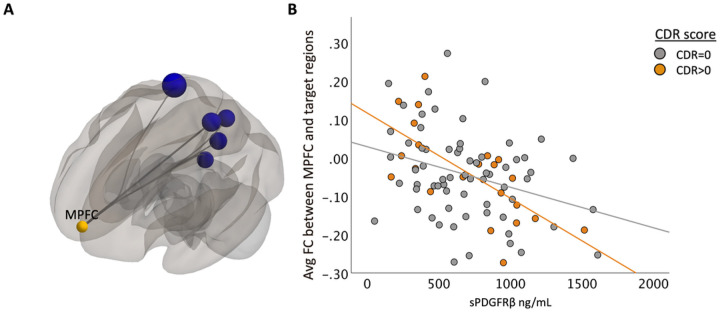
sPDGFRβValues Negatively Correlated with Resting-State Functional Connectivity using MPFC as seed region with uncorrected p-value. Panel A shows decreased FC between DMN seed region medial prefrontal cortex and ROIs within the parietal and frontal lobe are correlated with increased CSF sPDGFRβvalues projected on a glass brain. This data is plotted in Panel B which uses an avg FC between seed region MPFC and significant target region shown in panel A on the y axis and sPDGFRβ on the x axis. Patients with CDR scores 0.5 and above show a more marked decrease.

**Figure 4:**
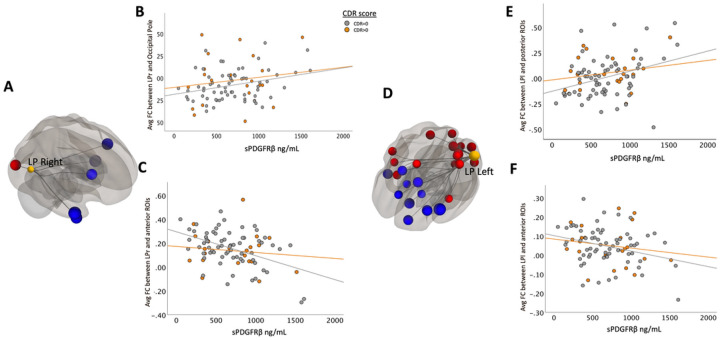
Bilateral parietal seed ROIs revealed both negative and positive correlations between sPDGFRβ values and target ROIs. Panels A and D show the significant FC between DMN bilateral parietal seed regions that are correlated with increased CSF sPDGFRβvalues projected on a glass brain. Significant posterior target brain regions were averaged and plotted in panels B and E by corresponding sPDGFRβon the x axis. Plotted data is stratified by color to appreciate how cognitive status affects FC’s relationship with sPDGFRβ(CDR scores above 0.5 coded in orange, CDR scores equaling 0 coded in grey).

**Figure 5:**
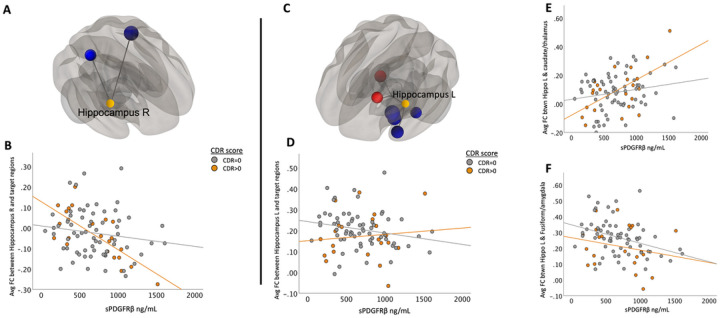
Correlations Between sPDGFRβ Values and Resting-State Functional Connectivity Differed Based on CDR Score when using hippocampus as seed region. The main effect of functional connectivity between right hippocampus and target ROIs in the superior temporal and parietal regions and sPDGFRβvalues was significant (*F*(1,84) =14.64, *p*<0.001, Panel A). A significant interaction effect was seen using CDR score as an interaction term showing patients with CDR score higher than zero had greater negative correlation between averaged significant FC and sPDGFRβvalues (*F*(1,84) = 6.15, *p*=0.015, Panel B). Left hippocampal seed region showed two clusters that exhibited both positive and negative correlations between FC and sPDGFRβ values (Panel C). Panel D shows the plots the averaged significant FC values (y-axis) against sPDGFRβvalues (x-axis). Panel E shows the positive correlation between CSF marker sPDGFRβ and FC values between the left hippocampus and caudate and thalamus (*F*(1,84)=13.16, *p*<0.001) showing a significant interaction with CDR score. Panel F shows the negative correlation between sPDGFRβand FC consisted of target regions in the fusiform gyrus, parahippocampus and amygdala region (Figure 5F, Table 2).

For exploratory analyses, a composite ROI was created by averaging the functional connectivity across all seeds and targets. Age and sex were included as model covariates with FDR corrected p-value set at 0.05. Additionally, an uncorrected p-value at 0.05 was also used for an exploratory analysis limiting the number of target ROIs (see supplemental material, Table 1 for complete list of target ROIs used). This approach revealed other potentially meaningful patterns that could be considered in future analyses. To understand how cognitive status (cognitively unimpaired vs. cognitively impaired) affected the relationship between sPDGFRβ values and resting-state functional connectivity, all data were plotted to visualize differences between cognitively normal individuals (colored in grey) versus those with mild cognitive impairment (colored in orange), Figs. 2–5 and tested for interaction effects using SPSS v28.

## Results

### Demographic Characteristics of the Cohort

A total of 89 participants (67 cognitively unimpaired, 22 cognitively impaired individuals) were included in this study. Of the total sample, 41 participants were male and 41 were *APOE4* carriers. Between cognitively unimpaired and impaired individuals, there was no difference in age, or CSF marker sPDGFRβ values. Results are summarized in Table 1.

### sPDGFRβ Values Negatively Correlated with Resting-State Functional Connectivity using PCC as seed region

Linear regression was used to assess the association between higher sPDGFRβ values and the functional connectivity strength among each DMN seed regions and all target pairwise ROIs (Table 2) at FDR-corrected p-value 0.05. Age and sex were used as covariates.

Using the DMN seed region posterior cingulate cortex (PCC), a significant negative correlation was found between sPDGFRβ and functional connectivity values. Specifically, the functional connectivity between PCC and both the left inferior frontal (R^2^ = 0.13, *t*(85)=−3.59, p = 0.047) and posterior cingulate gyrus (R^2^ = 0.135, *t*(85)=−3.52, p = 0.047) was lower as sPDGFRβ values increased (Fig. 2).

### Exploratory analysis using uncorrected p-value of 0.05

When using the MPFC as a seed region, there was a significant negative correlation between higher sPDGFRβ values and functional connectivity values between MPFC and 6 ROIs (Fig. 3) which include the precuneus, right cuneal cortex, posterior cingulate cortex (PCC), right lingual gyrus (rLG), right superior parietal lobule (rSPL), and right intracalcarine cortex (rICC).

Bilateral parietal seed ROIs revealed both negative and positive correlations between sPDGFRβ values and seed and target ROIs (listed in Table 2). When brain regions were divided via an anterior-posterior axis, different relationships with sPDGFRβ values were observed. FC between parietal seed regions and anterior regions correlated negatively to increased BBB breakdown whereas FC between parietal seed regions and posterior regions correlated positively to BBB breakdown (shown in Fig. 4, Table 2).

The main effect of functional connectivity between right hippocampus and target ROIs in the superior temporal and parietal regions and sPDGFRβ values was significant (*F*(1,84) = 14.64, *p* < 0.001, Fig. 5A–B). The left hippocampal seed region showed two clusters that exhibited both positive and negative correlations between FC and sPDGFRβ values. Specifically, there was found to be a positive correlation between CSF marker sPDGFRβ and FC values between the left hippocampus, caudate and thalamus (*F*(1,84) = 13.16, *p* < 0.001). The negative correlation between sPDGFRβ and FC consisted of target regions in the fusiform gyrus, parahippocampus, and amygdala region (Fig. 5F, Table 2).

### Correlations Between sPDGFRβ Values and Resting-State Functional Connectivity by CDR Score

When using seed regions PCC (Fig. 2C) and mPFC (Fig. 3B), visual indication of a more marked decrease between FC and sPDGFRβ values in patients with CDR scores 0.5 and above was observed, but there was no significant interaction using CDR score. The bilateral hippocampi regions exhibited an interaction effect. A significant interaction effect was observed for functional connectivity between right hippocampus and temporal/parietal regions and sPDGFRβ values when using CDR score as an interaction term, such that cognitively impaired patients (CDR > 0) had greater negative correlation between FC and sPDGFRβ values (*F*(1,84) = 6.15, *p* = 0.015, Fig. 5B). The left hippocampus had a significant interaction with CDR score such that a CDR score higher than zero had a greater positive correlation between functional connectivity between left hippocampus and thalamus and caudate and sPDGFRβ (*F*(1,84) = 13.16, *p* = 0.045, Fig. 5C,E). When the bilateral parietal regions were used as seed ROIs, no interaction effects were observed with CDR score.

**Figure 6:**
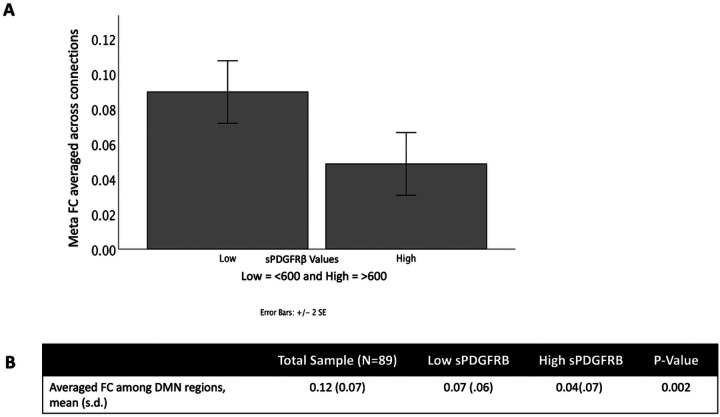
sPDGFRβValues Negatively Correlated with Resting-State Functional Connectivity overall. An overall decrease in functional connectivity using DMN seed regions and significant target regions was found when sPDGFRβ values were high (*t*(88)=10.13, *p*<0.001).

Across all participants, we found an overall decrease in functional connectivity using a composite ROI (which combined the functional connectivity across all seeds and targets) when sPDGFRβ values were high (*t*(88) = 10.13, *p* < 0.001, Fig. 6).

## Discussion

Previous studies have already shown that the loss of structural integrity of the BBB correlates with early cognitive dysfunction (Nation et al., 2019; Sengillo et al., 2013; Montagne et al., 2020) independent of Aβ and tau PET levels. However, it is unknown whether that structural breakdown of BBB is reflected within resting state functional connectivity. Resting state MRI can measure functional connectivity changes in the early stages of Alzheimer’s disease, particularly in brain areas known to be affected (i.e. the DMN). Therefore, to have a more complete understanding of the pathogenic role of BBB breakdown in the brain, it is important to investigate that relationship between vascular and functional activity changes in parts of the brain known to be adversely affected. Using CSF sPDGFRβ as a biomarker of BBB breakdown, we report that increased breakdown correlated with decreased functional connectivity in DMN areas, specifically the PCC.

The PCC is an important region of the traditional default mode network and plays important roles in episodic memory, spatial attention, self-evaluation, and other cognitive functions (Gusnard et al., 2001; Greicius et al., 2003; Ries et al., 2006; Braak and Braak 1991). Numerous studies have found diminished functional connectivity of the PCC and brain neocortex in patients with early AD and those carrying AD susceptible genes, suggesting that there is a reduced connectivity between the PCC and the medial temporal lobe, where initial histopathological changes occur in AD (Braak and Braak 1991). It is believed that injury to the medial temporal lobe directly affects the functional connectivity with the PCC, leading to decreased metabolic activity within the PCC (Wang et al., 2009). In addition, there is widespread loss of connectivity within the neocortex area of the brain. Zhong, et al. found that the PCC was a convergence center that received interactions from most other regions in the DMN, and it has been speculated that the PCC integrates the signals in the DMN and plays a role in memory identification, storage, and extraction (Miao et al., 2011). Additionally, Yu et al., found that outside of the DMN, functional connectivity between the PCC and left frontal gyrus were weakened in MCI patients. This is consistent with our findings that show lower functional connectivity between PCC and frontal and cingulate gyrus in participants with more BBB breakdown as measured by CSF sPDGFRβ.

Using DCE-MRI with gadolinium-based contrast agents, it was revealed that BBB breakdown occurs early in individuals with MCI and AD-type dementia as determined by the presence of gadolinium (an indicator of subtle BBB leaks) in the brain (Montagne et al., 2015; Nation et al., 2019). With the finding that lower functional connectivity associated with higher CSF sPDGFRβ, we further conclude that BBB permeability is associated with resting state FC, suggesting an association between BBB leakage and neural pathology in AD. To further this knowledge, we explored whether other DMN regions showed any similar results. Looking at uncorrected significant correlations between FC and CSF sPDGFRβ, we found many consistent results showing decreased functional connectivity between DMN regions correlating with increased BBB breakdown. Notably, bilateral parietal seed ROIs revealed both negative and positive correlations between sPDGFRβ values. These relationships were distinctly different along an anterior-posterior division such that FC between the lateral parietal and anterior regions correlated negatively to increased BBB breakdown and posterior regions correlated positively to BBB breakdown. This anterior-posterior division supports a disconnection syndrome due to the alterations of structural and functional integrity (Delbeuck et al., 2003). The disconnection in long distance FC between DMN hubs and the anterior brain area, which is significantly correlated with cognitive impairment (Liu et al., 2014) is another characteristic of AD (Zhang et al., 2009). One study found that cognitively impaired individuals demonstrated decreased FC in the posterior region of the retrosplenial cortex and more anterior prefrontal cortex (Yasuno et al., 2015). Like our findings, Tao and collegues (2020) found that there seemed to be a transitional stage of functional connectivity in the AD progression that presents as a disconnection between anterior and posterior brain regions among those individuals in the earliest stage of cognitive decline. It could be the case that we are seeing an early consequence of pathology that results in a very distinct disconnect between how the anterior and posterior portions of the brain communicate with each other. Ultimately, results are inconsistent but nevertheless we hope to continue to add knowledge to this field in the hopes of understanding the neurological mechanisms underlying cognitive decline, BBB breakdown and FC in the context of AD.

In fact, to have a clearer understanding on whether cognitive impairment drives this relationship between BBB breakdown and FC, all data were plotted to visualize differences between cognitively normal individuals (colored in grey) versus those with mild cognitive impairment (colored in orange) and tested for interaction effects which were only observed in the hippocampus. Using CDR score as an interaction term, we found that the relationship between sPDGFRβ and functional connectivity between right hippocampus and target ROIs in the superior temporal and parietal regions differed significantly compared to those individuals with CDR score of 0. This was also the case in looking at the relationship between sPDGFRβ and left hippocampus, and caudate and thalamus regions. While we had expected to find greater effects among our cognitively impaired group across multiple DMN brain regions, the fact that we saw them in only the hippocampus is consistent with previous work showing that BBB permeability is increased specifically in hippocampus but not in other regions including frontal and temporal cortex and other deep grey matter regions such as the thalamus and striatum (Nation et al, 2019). Considering that these findings are consistent with reporting that BBB breakdown during normal aging and MCI starts in the hippocampus (Montagne et al., 2015), our results add to this knowledge by incorporating functional connectivity and indicate that these measures results are sensitive to cognitive impairment.

The main limitation of the present study is a smaller sample size that was not adequately powered to stratify results by CDR and APOE4 carriers versus non carriers as well as an unequal sample of those that had a CDR score greater than zero. This was mainly due to matching MRI, CSF and CDR within 90 days of each other. For this reason, we wanted to expand our results to be more exploratory in hopes of directing future research. Additionally, given that this was not longitudinal data, it cannot be assumed that all individuals are on an AD trajectory and therefore this population may be heterogenous in disease/pathology.

## Conclusions

Ultimately, we conclude that breakdown that BBB breakdown as measured by CSF sPDGFRβ affects neural networks (most likely via disruptions in cerebral blood flow as shown previously) which results in decreased functional connections that lead to cognitive dysfunction.

## Data Availability

All imaging and demographic data are available in the Image and Data Archive (IDA) at Loni: https://ida.loni.usc.edu. All other data are available upon request from corresponding author.
